# The growing impact of older patients in the emergency department: a 5-year retrospective analysis in Brazil

**DOI:** 10.1186/s12873-020-00341-y

**Published:** 2020-06-11

**Authors:** João Carlos Pereira Gomes, Roger Daglius Dias, Jacson Venancio de Barros, Irineu Tadeu Velasco, Wilson Jacob Filho

**Affiliations:** 1 Emergency Department, Hospital das Clínicas da Faculdade de Medicina da Universidade de São Paulo, Av Dr. Enéas Carvalho de Aguiar, 255, 5° andar, s.5023, São Paulo, SP CEP: 05403–010 Brazil; 2 Department of Emergency Medicine, Harvard Medical School, Boston, MA USA; 3 STRATUS Center for Medical Simulation, Brigham and Women’s Hospital, Boston, MA USA; 4 Department of Information Technology, Hospital das Clínicas da Faculdade de Medicina da Universidade de São Paulo, São Paulo, SP Brazil; 5 Faculdade de Medicina da Universidade de São Paulo, São Paulo, SP Brazil; 6 Division of Geriatrics, Department of Internal Medicine, Hospital das Clínicas da Faculdade de Medicina da Universidade de São Paulo, São Paulo, SP Brazil

**Keywords:** Aged demography, Emergency department, Older, Outcome, Routinely collected data

## Abstract

**Background:**

The average age of the global population is rising at an increasing rate. There is a disproportional increase in Emergency Department (ED) visits by older people worldwide. In the Brazilian health system, complex and severely ill patients and those requiring specialized urgent procedures are referred to tertiary level care. As far as we know, no other study in Latin America has analyzed the impact of demographic changes in tertiary ED attendance. Aim: To describe the sociodemographic characteristics and outcomes of tertiary Brazilian ED users.

**Methods:**

Design: Observational cross-sectional analytic study. Setting: Emergency Department, tertiary university hospital, São Paulo, Brazil. Participants: patients aged 18 years or older attending a tertiary ED (2009–2013). The primary outcomes were hospitalization and mortality; the secondary outcome was ICU admission. Age was categorized as ‘young adults’ (18-39y), ‘adults’ (40-59y), ‘young-older adults’ (60-79y), and ‘old-older adults’ (80-109y). Other variables included sex, reason for attendance, time of ED visit, mode of presentation, type of hospitalization, main procedure, length of hospital stay (LOS) and length of ICU stay (ICU-LOS). We calculated descriptive statistics, built generalized linear mixed models for each outcome and estimated Odds Ratios (95% CI) for the independent categorical variables. The significance level was 5% with Bonferroni correction.

**Results:**

Older age-groups represented 26.6% of 333,028 ED visits, 40.7% of admissions, 42.7% of ICU admissions and 58% of all deaths. Old-older patients accounted for 5.1% of ED visits, 9.5% of admissions and 10.1% of ICU admissions. Hospitalization, ICU admission and mortality rates increased with older age in both sexes. LOS and ICU-LOS were similar across age-groups. The proportions of visits and admissions attributed to young adults decreased annually, while those of people aged 60 or over increased. The ORs for hospitalization, ICU admission and mortality associated with the old-older group were 3.49 (95% CI = 3.15–3.87), 1.27 (1.15–1.39) and 5.93 (5.29–6.66) respectively, with young adults as the reference.

**Conclusions:**

In tertiary ED, age is an important risk factor for hospitalization and mortality, but not for ICU admission. Old-older people are at the greatest risk and demand further subgroup stratification.

## Background

The average age of the global population is rising at an increasing rate. People aged 60 and over accounted for 13% of the global population in 2017. While this age group is likely to double by 2050, the population younger than 15 is expected to remain stable [1].

Although older people are a heterogenous group in terms of physiological reserve and rate of functional decline, multimorbidity and use of health services tend to increase as age rises [2–4]. The increase in emergency department (ED) visits by older individuals is greater than the rate of growth of this population in North America, Europe, Asia and Oceania [5–8]. Furthermore, compared to younger adults, older ED patients on average have earlier ED returns, longer hospital stays, greater resource use, and higher rates of hospitalization and adverse outcomes [9, 10].

In the Brazilian health system, complex and severely ill patients and those requiring specialized urgent procedures are referred to tertiary level care [11]. As far as we know, no other study in Latin America has analyzed the impact of demographic changes in tertiary ED attendance.

### Aim

To describe the demographic profile of ED users in a tertiary hospital over a 5-year period and to investigate differences in outcomes by sex and age.

## Methods

### Design

Observational cross-sectional analytic study.

### Study period

1st January 2009 to 31st December 2013.

### Setting

São Paulo, capital of São Paulo State is the largest city in Brazil, with an estimated population of 12 millions of people. Hospital das Clínicas (HC) is a teaching hospital complex with 2200 beds of University of São Paulo Medical School, serving as the referral center for the whole state. The Central Institute, a tertiary university hospital, is the main unit within the Hospital das Clínicas complex.

### Participants

#### Eligibility criteria

We considered for inclusion all patients aged 18 years or older attending the ED associated with the Hospital das Clínicas Central Institute (ED-HC). Those with obstetric, ophthalmological or otolaryngological problems were not eligible, since they were treated in the adjacent unit. We excluded records if they were incomplete or inconsistent, or if there were duplicates of the unique hospital attendance number or admission authorization form number. Furthermore, we excluded cases that resulted in hospital transfers; outcomes other than discharge or admission and patients that left without a medical consultation (LWBS) or against medical advice (LAMA).

To be eligible for inclusion, a medical evaluation had to be completed during the visit, and its outcome recorded as either admission or discharge. In addition, when the ED visit resulted in admission, we only included those with a final outcome coded as discharge or death. We excluded all other types of ED attendance and admission.

#### Participant selection

On arrival at ED-HC, the patient (or those accompanying them) provides their personal data and reason for attendance which are recorded in the electronic registration. The system generates a unique number for each separate ED-HC attendance. We considered an ED visit to be complete if a medical evaluation was finished and an outcome recorded electronically (discharge, admission, hospital transfer or other). Patients requiring more than 12 h of observation were admitted.

For each admission, the responsible doctor fills out an admission authorization form with the patient’s data; this generates a new admission authorization form number for the billing system [12, 13]. To conclude the admission, the doctor has to select the main ICD-10 code as well as the outcome (discharge, death, hospital transfer, self-discharge or other). If the patient dies in ED, it is standard practice to admit them on the system with the outcome coded as death.

### Variables

We analyzed eligible ED-HC visits with respect to the following variables: age and sex of the patient; year of attendance; mode of presentation to ED-HC being either ‘spontaneous’ (without prior evaluation by another service) or ‘referred’ (having already accessed a different health service, and arriving by ambulance or helicopter); time of ED visit (day shift 7 AM-7 PM or night shift 7 PM-7 AM); and ED outcome (admission or discharge). In cases resulting in admission we analyzed additional factors. These were type of hospitalization (surgical, clinical or other); main procedure (surgical, clinical or transplant-related); length of hospital stay (LOS); use or not of the ICU; length of ICU stay (ICU-LOS); and final admission\ outcome (discharge or death).

The categorical variables were the following: age-group, year of presentation, mode of presentation, reason for attendance, time of ED visit, type of hospitalization, main procedure, and ICU admission. We stratified age into the following groups: young adults (18–39 years), adults (40–59), young-older adults (60–79) and old-older adults (80–109). We categorized the reasons for attendance as either ‘external causes’ (injuries or health conditions related to accidents, trauma, burns, poisoning, environmental events and others, either unintentional or intentional), ‘general and localized symptoms’, ‘evaluation requested by another service’, ‘scheduled attendances’, or ‘other’. The continuous variables were age, LOS (the interval between the admission and discharge billing dates), and ICU-LOS (ICU days billed). Furthermore, we stratified LOS into six categories: 0–1, 2, 3, 4–7, 8–20, > = 21 days of hospitalization. Hospital admissions lasting one day or less were grouped as one category (0–1).

The primary dichotomous outcomes were hospitalization (admission vs ED discharge), and mortality (death vs hospital discharge). The secondary dichotomous outcome was ICU admission (or not). The primary aim was to investigate associations between demographic characteristics (age and sex) and the outcome variables.

### Data source

We retrieved routinely collected data from administrative electronic registers maintained by HC, then consolidated them to produce a single dataset. ED attendance data are recorded in the hospital information system, and admissions data in the hospital billing system. In some cases, it was necessary to recode entries, depending on how they were recorded in the electronic system. Otherwise, we obtained data directly from the hospital databases.

### Potential biases and analytic issues

This is an observational analytic study of electronic health data collected routinely for administrative purposes and for documentation of clinical care. Routinely collected health data are defined as those collected without a pre-existing research question [14]. Guidelines such as STROBE and its extension, RECORD (REporting of studies Conducted using Observational Routinely-collected health Data), were developed to enhance the quality of observational research and the transparency of results [15, 16]. We used the STROBE and RECORD statements as reporting guidelines.

The study covers a five-year period, and some patients had multiple ED visits and admissions. We identified individuals with more than one ED visit, ranking them by total number of attendances over the 5-year period. As such, we determined an upper-limit for inclusion in the study.

The reasons for attendance were varied and numerous; 72 were recorded in the hospital information system. To facilitate the analysis, we assigned broader categories (‘external causes’, ‘general and localized symptoms’, ‘evaluation requested by another service’, ‘scheduled attendances’, or ‘other’). The category of ‘scheduled attendance’, which describes non-emergency visits (e.g. returning for test results), represents neither ‘spontaneous’ nor ‘referred’ modes of ED presentation (see Variables in the main text), and was therefore defined as *missing* data.

During the study period, there were changes in the triage processes at ED-HC. Manchester Triage System version II, a new triage system based on individual clinical risk was implemented [17]. We analyzed year of attendance and mode of presentation in order to identify any effect due to these changes.

The high number of study subjects (ED visits) demands a measure of effect size, such as an Odds Ratio (OR) (or log OR), to estimate the magnitude of effect or association between two or more variables [18–20]. The effect size together with its confidence interval provides an estimate of the magnitude of an effect of interest and the precision of that estimate [21, 22]. Generalized linear mixed models (GLMM) for a given dichotomous outcome (dependent variable), using binomial probability distribution and logit link function, allow an estimate of ORs (with 95% confidence intervals) for independent variable categories in relation to respective reference categories.

### Statistical analysis

We calculated summary statistics for ED-HC visits and admissions. Categorical variables are presented as total count (n) and percentage. Continuous variables are presented as mean (standard deviation) or median (maximum and minimum values). Descriptive statistics were further stratified according to year, sex and age-group. For the multivariate analysis, generalized linear mixed models were built in order to investigate variables associated with the primary and secondary dichotomous outcomes. All three models had binomial probability distribution and logit link function Results are presented as odds ratios (OR) with 95% confidence intervals (CI). The significance level was set at 5% with Bonferroni correction. All analyses were conducted using SPSS Statistics version 25.0 (IBM, Corp., Armonk, NY).

## Results

### Participants

**Fig. 1:**
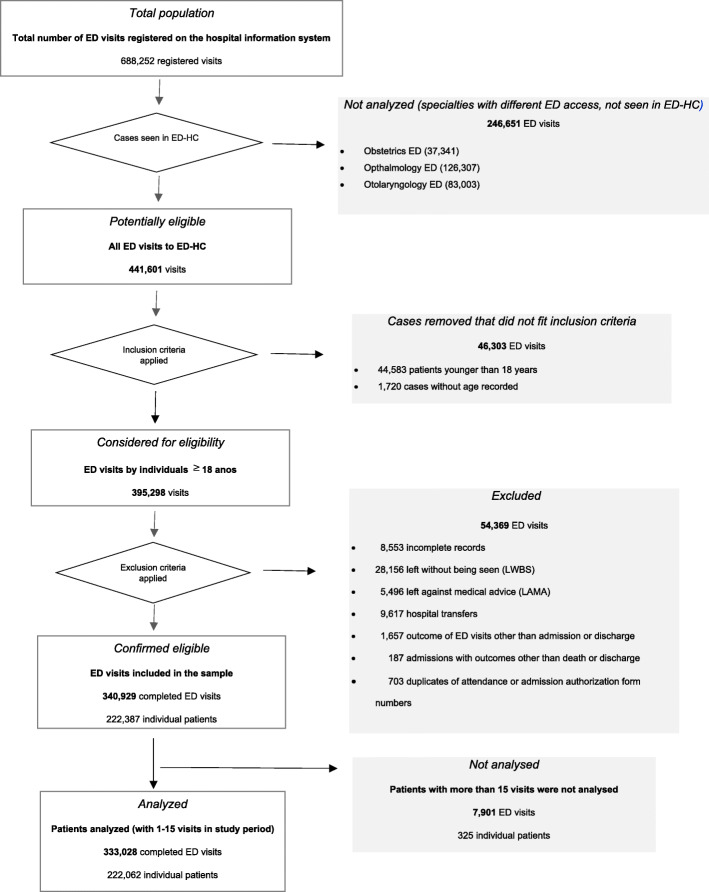
Flow-diagram of case selection process

Figure 1 shows the selection process for the study population. After exclusions, the eligible sample was made up of 340,929 consecutive attendances, associated with 222,387 individual patients. The mean (SD) number of visits per person over the 5-year period was 1.53 (1.60) with a range of 1 to 136. The 25th, 50th and 75th percentiles were 1.00, 1.00 and 2.00 respectively. We set a cut-off point for inclusion in the analysis at 15 ED visits per person. Applying this value, there were 222,060 patients (99.99% of the sample) with between 1 and 15 visits, resulting in 333,028 complete attendances (97.68% of the sample). The mean (SD) age was 46.7 (18.6) and the median was 45, with a range of 18 to 108 years (See Table 1).

**Table 1:** Sample selection for analysis

	ED visits/patient	ED visits, N	(%)	patients, N	(%)	mean	(SD)	median	(min-max)
Eligible sample	1 to 15	333,028	(97.68)	222,062	(99.999)	46.7	(18.6)	45.0	(18–108)
	1	166,364	(48.80)	166,364	(74.92)	44.1	(18.2)	41.0	(18–108)
	2 to 15	166,664	(48.88)	55,698	(25.08)	49.2	(18.7)	48.0	(18–103)
	> 15	7901	(2.32)	325	(0.001)	54.9	(18.3)	55.0	(20–99)

*SD* Standard Deviation, *min-max* minimum-maximum. Mean, SD, median and min-max refer to age in years

### Characteristics of study subjects

Women made up over half the ED visits (54.6%). Young adults accounted for the majority of attendances (40.8%), followed by adults (32.7%), young-older adults (21.5%) and old-older adults (5.1%). Between 2009 and 2013, the mean and median ages increased by 3.2 and 4 years, respectively (Table 2). There were 52,592 admissions (15.8% of ED attendances), with 13,615 requiring an ICU stay (25,9% of admissions). Of 52,592 hospitalizations, 6674 resulted in death, giving a mortality rate of 12.7% (Table 3).

**Table 2:** ED visit demographic data

	ED visits N	(%)	mean age	(SD)	median age	(min-max)
2009	71,833	(21.6)	45.2	(18.5)	43	(18–103)
2010	68,386	(20.5)	46.1	(18.3)	44	(18–104)
2011	70,715	(21.2)	46.8	(18.3)	46	(18–101)
2012	76,224	(22.9)	47.4	(18.6)	47	(18–108)
2013	45,870	(13.8)	48.4	(19.4)	47	(18–107)
**Total**	**333,028**	**(100.0)**	**467**	**(186)**	**45**	**(18–108)**
Women	181,884	(54.6)	46.5	(18.6)	45	(18–108)
Men	151,144	(45.4)	46.9	(18.5)	46	(18–102)
young adults	135,763	(40.8)	28.4	(6.1)	28	(18–39)
adults	108,830	(32.7)	49.5	(5.7)	50	(40–59)
young-older adults	71,602	(21.5)	68.2	(5.6)	68	(60–79)
old-older adults	16,833	(5.1)	84.7	(4.1)	84	(80–108)

Percentages are out of the total number of eligible ED visits, or total number of admissions, after exclusions. *SD* Standard Deviation, *min-max* minimum-maximum. Mean, SD, median and min-max refer to age in years

**Table 3:** ED visit characteristics and missing data

ED visit Variables	categories	N	(% ED visits)	% Valid
Mode of presentation	Spontaneous	316,153	(94.9)	94.9
	Referred	16,875	(5.1)	5.1
	**Valid**	**333,028**	**(100.0)**	**100.0**
	*Missing*	0		
Time of ED visit	Day shift (7 AM-7 PM)	246,520	(74.0)	74.0
	Night shift (7 PM-7 AM)	86,508	(26.0)	26.0
	**Valid**	**333,028**	**(100.0)**	**100.0**
	*Missing*	0		
Reason for attendance	Local symptoms	106,831	(32.1)	32.8
	General symptoms	150,423	(45.2)	46.2
	External causes	30,323	(9.1)	9.3
	Evaluation	37,780	(11.3)	11.6
	**Valid**	**325,357**	**(97.7)**	**100.0**
	*Missing* (scheduled visits)	7671	(2.3)	
ED outcome	Admission	52,592	(15.8)	15.8
	Discharge	280,436	(84.2)	84.2
	**Valid**	**333,028**	**(100.0)**	**100.0**
	*Missing*	0		
**Admission Variables**				
Type of hospitalization	Surgical	26,091	(7.8)	50.5
	Clinical	25,537	(7.7)	49.5
	**Valid**	**51,628**	**(15.5)**	**100.0**
	*Missing* (other specialties)	964	(0.3)	
	*Missing* (discharged from ED)	280,436	(84.2)	
Main Procedure	Clinical	36,376	(10.9)	69.4
	Surgical	14,564	(4.4)	27.8
	Transplant-related	1493	(0.4)	2.8
	**Valid**	**52,433**	**(15.7)**	**100.0**
	*Missing* (diagnostic testing)	146	(0.0)	
	*Missing* (discharged from ED)	280,449	(84.2)	
Use of ICU	No	38,977	(11.7)	74.1
	Yes	13,615	(4.1)	25.9
	**Valid**	**52,592**	**(15.8)**	**100.0**
	*Missing* (discharged from ED)	280,436	(84.2)	
Final admission outcome	Death	6674	(2.0)	12.7
	Discharge	45,918	(13.8)	87.3
	**Valid**	**52,592**	**(15.8)**	**100.0**
	*Missing* (discharged from ED)	280,436	(84.2)	
Categorized LOS	0–1	11,252	(3.4)	21.4
	2–2	6455	(1.9)	12.3
	3–3	4612	(1.4)	8.8
	4–7	11,158	(3.4)	21.2
	8–20	13,462	(4.0)	25.6
	21+	5640	(1.7)	10.7
	**Valid**	**52,579**	**(15.8)**	**100.0**
	*Missing* (discharged from ED)	280,449	(84.2)	

*Missing data*: 7671 (2.3%) due to ‘scheduled attendances’, 964 (0.3%) due to admissions to other specialties and 146 (0.0%) due to diagnostic testing. Valid percentages are out of the total number of eligible ED visits, or total number of admissions, after exclusions and without missing data

Table 4 presents ED visit and admission data stratified by age-group and sex. Older age-groups were responsible for 26.6% of ED visits, 37% of referred presentations, 40.8% of all admissions, 42.7% of ICU admissions and 58.1% of all deaths. There were a greater number of men that attended with a referred presentation. Proportionally more men than women were admitted (18.6% vs 13.4%), and they carried a higher in-patient mortality rate (13.4% vs 11.9%). The number of referred presentations, admissions, and in-patient deaths increased proportionally with advancing age from young to old-older adults.

**Table 4:** ED visit and admission data categorized by sex and age-group

	**Women**	(%)	**Men**	(%)	**Total (100%)**	**young-adult**	(%)	**adult**	(%)	**young-older adult**	(%)	**old-older adult**	(%)	**Total (100%)**
ED visits N (%)	181,884	(54.6)	151,144	(45.4)	**333,028**	135,763	(40.8)	108,830	(32.7)	71,602	(21.5)	16,833	(5.1)	**333,028**
Referred presentation N (%)	6808	(40.3)	10,067	(59.7)	**16,875**	5473	(32.4)	5144	(30.5)	4428	(26.2)	1830	(10.8)	**16,875**
Admissions N (%)	24,429	(46.5)	28,163	(53.5)	**52,592**	13,708	(26.1)	17,448	(33.2)	16,464	(31.3)	4972	(9.5)	**52,592**
ICU admissions N (%)	6194	(45.5)	7421	(54.5)	**13,615**	3343	(24.6)	4455	(32.7)	4441	(32.6)	1376	(10.1)	**13,615**
Deaths N (%)	2914	(43.7)	3760	(56.3)	**6674**	914	(13.7)	1884	(28.2)	2630	(39.4)	1246	(18.7)	**6674**
	**Women**	(%)	**Men**	(%)	*p-value*	**young-adult**	(%)	**adult**	(%)	**young-older adult**	(%)	**old-older adult**	(%)	*p-value*
**ED visit characteristics**														
Referred presentation N (%)	6808	(3.7)	10,067	(6.7)	*< .001*	5473	(4.0)	5144	(4.7)	4428	(6.2)	1830	(10.9)	*< .001*
Admission N (%)	24,429	(13.4)	28,163	(18.6)	*< .001*	13,708	(10.1)	17,448	(16.0)	16,464	(23.0)	4972	(29.5)	*< .001*
**Total ED visits N (%)**	**181,884**	**(1000)**	**151,144**	**(100.0)**		**135,763**	**(100.0)**	**108,830**	**(100.0)**	**71,602**	**(100.0)**	**16,833**	**(100.0)**	
**Admission characteristics**														
Surgical hospitalization N (%)	11,969	(50.1)	14,122	(50.9)	*.052*	7147	(53.2)	8665	(50.6)	8043	(49.7)	2236	(45.6)	*< .001*
Surgical main procedure N (%)	6683	(27.4)	7881	(28.1)	*.104*	3963	(29.0)	4809	(27.7)	4536	(27.6)	1256	(25.3)	*.014*
ICU admissions N (%)	6194	(25.4)	7421	(26.4)	*.009*	3343	(24.4)	4455	(25.5)	4441	(27.0)	1376	(27.7)	*< .001*
Deaths N (%)	2914	(11.9)	3760	(13.4)	*< .001*	914	(6.7)	1884	(10.8)	2630	(16.0)	1246	(25.1)	*< .001*
**Total hospital admissions**	**24,429**	**(100.0)**	**28,163**	**(100.0)**		**13,708**	**(100.0)**	**17,448**	**(100.0)**	**16,464**	**(100.0)**	**4972**	**(100.0)**	
**Length of stay**														
ICU-LOS mean (SD)	6.6 (8.0)		6.7 (8.0)			6.3 (7.7)		6.7 (7.9)		6.9 (8.3)		6.8 (8.1)		
ICU-LOS median (max-min)	4.0 (1–74)		4.0 (1–90)			3.0 (1–71)		4.0 (1–90)		4.0 (1–68)		4.0 (1–74)		
LOS mean (SD)	8.6 (11.5)		8.6 (10.9)			8.3 (10.7)		8.7 (11.0)		8.7 (11.8)		8.7 (11.1)		
LOS median (max-min)	5.0 (0–490)		5.0 (0–178)			4.0 (0–125)		5.0 (0–184)		5.0 (0–490)		5.0 (0–193)		

*LOS* length of hospital stay, *ICU-LOS* length of ICU stay, *SD* Standard Deviation, *min-max* minimum-maximum. Mean, SD, median and min-max refer to LOS and ICU-LOS in days. Percentages are out of the total number of eligible ED visits, or total number of admissions

Amongst old-older adults, the rates of admission (29.5%) and of in-hospital mortality (25.2%) were three and four times that of young adults (10.1 and 6.7%, respectively). Old-older adults had proportionally fewer surgical hospitalizations and surgical procedures than other age-groups. The proportion of admitted patients requiring an ICU stay was similar for the young and adult groups, and again between young-older and old-older groups. LOS and ICU-LOS differed minimally according to age and sex (Table 4).

Table 5 presents the same data, stratified by sex and age-group combined. There were more female ED attendees in every age bracket. Admission and mortality rates increased with age across both sexes. In all age-groups except old-older adults, there were more men than women with referred presentations, with more men requiring hospital admission, surgical hospitalization, surgical procedures and ICU stays. Mortality was also higher amongst men.

**Table 5:** ED visit and admission data by sex and age-group combined

Sex		Women				Men			
**Age-group**		young adult	adult	young-older adult	old-older adult	young adult	adult	young-older adult	old-older adult
Age-group/sex	N	74,658	60,590	36,886	9750	61,105	48,240	34,716	7083
	(%)	(41.0)	(33.3)	(20.3)	(5.4)	(40.4)	(31.9)	(23.0)	(4.7)
**ED visit characteristics**									
Referred presentations	N	1819	1955	1979	1055	3654	3189	2449	775
	(%)	(2.4)	(3.2)	(5.4)	(10.8)	(6.0)	(6.9)	(7.1)	(10.9)
Admissions	N	6217	7711	7631	2870	7491	9737	8833	2102
	(%)	(8.3)	(12.7)	(20.7)	(29.4)	(12.3)	(20.2)	(25.4)	(29.7)
Total ED visits	N	74,658	60,590	36,886	9750	61,105	48,240	34,716	7083
	(%)	(100.0)	(100.0)	(100.0)	(100.0)	(100.0)	(100.0)	(100.0)	(100.0)
**Admission characteristics**									
Surgical hospitalization	N	3169	3778	3731	1291	3978	4887	4312	945
	(%)	(52.4)	(50.1)	(49.9)	(45.6)	(53.9)	(51.0)	(49.6)	(45.6)
Surgical main procedure	N	1726	2151	2095	711	2237	2658	2441	545
	(%)	(27.8)	(28.0)	(27.5)	(24.9)	(29.9)	(27.4)	(27.7)	(26.0)
ICU admissions	N	1426	1926	2064	778	1917	2529	2377	598
	(%)	(22.9)	(25.0)	(27.0)	(27.1)	(25.6)	(26.0)	(26.9)	(28.4)
Deaths	N	288	715	1190	721	626	1169	1440	525
	(%)	(4.6)	(9.3)	(15.6)	(25.1)	(8.4)	(12.0)	(16.3)	(25.0)
Total admissions	N	6217	7711	7631	2870	7491	9737	8833	2102
	(%)	(100.0)	(100.0)	(100.0)	(100.0)	(100.0)	(100.0)	(100.0)	(100.0)
**Continuous variables**									
Age (years)	mean (SD)	28.3 (6.1)	49.4 (5.6)	68.2 (5.7)	84.9 (4.3)	28.5 (6.0)	49.6 (5.8)	68.2 (5.6)	84.3 (3.9)
	median (min-max)	28 (18–39)	49 (40–59)	68 (60–79)	84 (80–108)	28 (18–39)	50 (40–59)	68 (60–79)	83 (80–102)
ICU-LOS (days)	mean (SD)	6.2 (7.8)	6.6 (7.8)	6.9 (8.4)	6.8 (7.9)	6.3 (7.7)	6.7 (8.0)	6.8 (8.2)	6.9 (8.5)
	median (min-max)	3 (1–71)	4 (1–60)	4 (1–67)	4 (1–74)	3 (1–59)	4 (1–90)	4 (1–68)	4 (1–63)
LOS (days)	mean (SD)	8.3 (10.6)	8.7 (10.9)	8.8 (12.9)	8.5 (11.1)	8.3 (10.8)	8.6 (11.1)	8.7 (10.8)	9.0 (11.0)
	median (min-max)	4 (0–125)	5 (0–184)	5 (0–490)	5 (0–193)	4 (0–115)	5 (0–178)	5 (0–106)	5 (0–87)

*LOS* length of hospital stay, *ICU-LOS* length of ICU stay, *SD* Standard Deviation; min-max: minimum-maximum. Mean, SD, median and min-max refer to age in years and to LOS and ICU-LOS in days. Percentages are out of the total number of eligible ED visits, or total number of admissions

Table 6 presents data categorized by year of attendance. The proportion of ED-HC visits by young adults decreased annually, falling from 44.4 to 38.2% between 2009 and 2013. In contrast, people over 60 years accounted for proportionally more attendances, rising from 24.1 to 29.9%. Similar trends were observed for admissions and ICU admissions. In 2013, the number of ED visits fell significantly in all age groups, except amongst the old-older adult group. However, the total number of hospital and ICU admissions remained relatively stable. There was little variation in LOS during the 5-year period, but there was a reduction in the mean ICU-LOS.

**Table 6:** ED demographic, visit and admission data categorized by year of attendance

Year	2009		2010		2011		2012		2013	
	N	(%)	N	(%)	N	(%)	N	(%)	N	(%)
**ED visits demographic characteristics**										
Women	38,530	(53.6)	37,410	(54.7)	38,816	(54.9)	42,612	(55.9)	24,516	(53.4)
young adults	31,918	(44.4)	28,581	(41.8)	27,980	(39.6)	29,753	(39.0)	17,531	(38.2)
adults	22,583	(31.4)	22,520	(32.9)	24,387	(34.5)	24,724	(32.4)	14,616	(31.9)
young-older adult	14,236	(19.8)	14,115	(20.6)	14,922	(21.1)	17,997	(23.6)	10,332	(22.5)
old-older adults	3096	(4.3)	3170	(4.6)	3426	(4.8)	3750	(4.9)	3391	(7.4)
**ED visits characteristics**										
Referred visits	2995	(4.2)	3142	(4.6)	3696	(5.2)	3623	(4.8)	3419	(7.5)
Admissions	11,064	(15.4)	10,872	(15.9)	10,961	(15.5)	10,340	(13.6)	9355	(20.4)
**Total ED visits**	**71,833**	**(100.0)**	**68,386**	**(100.0)**	**70,715**	**(100.0)**	**76,224**	**(100.0)**	**45,870**	**(100.0)**
**Admissions demographic characteristics**										
Women	5043	(45.6)	5041	(46.4)	5102	(46.5)	4854	(46.9)	4389	(46.9)
young adults	3049	(27.6)	2945	(27.1)	2773	(25.3)	2606	(25.2)	2335	(24.9)
adults	3679	(33.2)	3647	(33.5)	3812	(34.7)	3327	(32.2)	2983	(31.9)
young-older adult	3401	(30.7)	3275	(30.1)	3328	(30.4)	3479	(33.6)	2981	(31.9)
old-older adults	935	(8.4)	1005	(9.2)	1048	(9.6)	928	(9.0)	1056	(11.2)
**Admissions characteristics**										
Surgical hospitalization	5052	(46.6)	5184	(48.6)	5274	(49.0)	5551	(54.7)	5030	(54.5)
Surgical main procedure	3074	(27.9)	2968	(27.4)	2871	(26.3)	2947	(28.6)	2704	(29.0)
ICU admissions	2479	(22.4)	2856	(26.3)	2782	(25.4)	2847	(27.5)	2651	(28.3)
Deaths	1240	(11.2)	1379	(12.7)	1407	(12.8)	1378	(13.3)	1270	(13.6)
**Total hospital admissions**	**11,064**	**(100.0)**	**10,872**	**(100.0)**	**10,961**	**(100.0)**	**10,340**	**(100.0)**	**9355**	**(100.0)**
**ICU admissions demographic characteristics**										
Women	1078	(43.5)	1300	(45.5)	1246	(44.8)	1305	(45.8)	1265	(47.7)
young adults	686	(27.7)	710	(24.9)	659	(23.7)	690	(24.2)	598	(22.6)
adults	815	(32.9)	923	(32.3)	996	(35.8)	895	(31.4)	826	(31.2)
young-older adult	770	(31.0)	912	(31.9)	863	(31.0)	987	(34.7)	909	(34.2)
old-older adults	208	(8.4)	311	(10.9)	264	(9.5)	275	(9.7)	318	(12.0)
**Total ICU admissions**	**2479**	**(100.0)**	**2856**	**(100.0)**	**2782**	**(100.0)**	**2847**	**(100.0)**	**2651**	**(100.0)**
**Length of stay**										
ICU-LOS mean (SD)	7.6 (9.4)		7.2 (8.6)		6.8 (8.2)		6.1 (7.2)		5.7 (6.3)	
ICU-LOS median (max-min)	4.0 (1–77)		4.0 (1–90)		4.0 (1–74)		4.0 (1–71)		4.0 (1–68)	
LOS mean (SD)	8.6 (11.3)		8.6 (11.1)		8.6 (10.9)		8.4 (11.4)		8.9 (11.4)	
LOS median (max-min)	4.0 (0–125)		4.0 (0–109)		5.0 (0–193)		5.0 (0–490)		5.0 (0–368)	

*LOS* length of hospital stay, *ICU-LOS* length of ICU stay, *SD* Standard Deviation, *min-max* minimum-maximum. Mean, SD, median and min-max refer to LOS and ICU-LOS in days. Percentages are out of the total number of eligible ED visits, or total number of admissions

### Main results

The main factors associated with hospital admission were referred presentation (OR 6.34); belonging to the old-older (3.49), young-older (2.70) and adult (1.75) age-groups; and male sex (1.37).

Admission to ICU was more frequent amongst patients with referred presentations (OR 1.36), and those with a surgical main procedure (OR 5.28). It was also more frequent among old-older (1.27), young-older (1.19) and adult (1.08) age-groups. The OR associated with the adult age-group (compared to young adults) did not reach significance of *p* < 0.007 with Bonferroni correction (*p* = 0.014). In a post-hoc analysis young-older and old-older adults also had a similar risk of ICU admission (*p* = 0.17). There was no association between sex and admission to ICU (*p* = 0.09).

The main risk factors associated with mortality were ICU admission (OR 7.34) and older age: old-older adults (5.93), young-older adults (3.41), and adults (2.04). External causes (2.26), presentation following referral (1.89) and male sex (1.14) also carried increased OR for mortality. There was no difference in mortality between the years analyzed (*p* = 0.59). Table 7 summarizes the multivariate analysis results.

**Table 7:** Generalized Linear Mixed Models main results

	Hospitalization				Admission to ICU				Mortality			
Fixed effect	***p***-value	Categories	OR	(CI 95%)	***p***-value	Categories	OR	(CI 95%)	***p***-value	Categories	OR	(CI 95%)
Year	< .001	2013	1.21	(1.12–1.32)	< .001	2013	1.31	(1.22–1.42)	.599	2013	1.03	(0.93–1.14)
		2012	0.80	(0.74–0.86)		2012	1.34	(1.24–1.44)		2012	1.07	(0.97–1.18)
		2011	0.94	(0.87–1.02)		2011	1.21	(1.12–1.31)		2011	1.05	(0.95–1.16)
		2010	1.01	(0.94–1.09)		2010	1.32	(1.22–1.42)		2010	1.08	(0.97–1.19)
		2009	ref			2009	ref			2009	ref	
Sex	< .001	men	1.37	(1.30–1.44)	.096	men	1.04	(0.99–1.09)	< .001	men	1.14	(1.07–1.21)
		women	ref			women	ref			women	ref	
Age-groups	< .001	old-older	3.49	(3.15–3.87)	< .001	old-older	1.27	(1.15–1.39)	< .001	old-older	5.93	(5.29–6.66)
		young-older	2.7	(2.52–2.88)		young-older	1.19	(1.11–1.27)		young-older	3.41	(3.09–3.76)
		adult	1.75	(1.64–1.86)		adult	1.08	(1.02–1.16)		adult	2.04	(1.84–2.25)
		young adult	ref			young adult	ref			young adult	ref	
Mode of presentation	< .001	referred	6.34	(5.81–6.91)	< .001	referred	1.36	(1.27–1.46)	< .001	referred	1.83	(1.71–2.01)
		self-initiated	ref			self-initiated	ref			self-initiated	ref	
Time of ED visit	<.001	Night shift	1.34	(1.27–1.42)	.031	Night shift	1.06	(1.01–1.12)	.004	Night shift	1.10	(1.03–1.17)
		Day shift	ref			Day shift	ref			Day shift	ref	
Type of hospitalization					<.001	Surgical	0.87	(0.81–0.92)	<.001	Surgical	0.64	(0.59–0.69)
						Clinical	ref			Clinical	ref	
Main procedure					<.001	Transplant	0.51	(0.43–0.61)	<.001	Transplant	0.59	(0.50–0.78)
						Surgical	5.28	(4.97–5.61)		Surgical	0.79	(0.73–0.86)
						Clinical	ref			Clinical	ref	
ICU admission									< .001	Yes	7.34	(6.75–7.97)
										No	ref	

*OR* Odds Ratio, *CI 95%* confidence interval 95%, *ref*. reference

## Discussion

In this cross-sectional observational study of secondary data, we demonstrate an association between older age and higher rates of referred presentation, hospital admission and mortality in a tertiary Brazilian ED. These results are consistent with existing literature. Older adults presenting to ED are often acutely ill and more likely to require higher-level care [23, 24]. A review of 11 international studies reported that one-third to one-half of all ED attendances by older adults resulted in hospital admission, with rates of hospitalization between 2.5 and 4.6 times higher than the youngest patients [25]. However, none of these studies included Latin America data.

Our results may indicate a shift towards an older demographic in the Brazilian ED population. There was an annual fall in the proportion of ED visits by young adults, with those over 60 accounting for proportionally more attendances each year. Recent national data corroborated this trend in the use of hospital resources [26].

Women account for the majority of people visiting ED. [27] Even in countries with greater healthcare utilization amongst men, there is a marked female predominance in the oldest ED patients [28–30]. In this study there were more women in all age-groups, with the exception of young-older adults. There was an increased chance of admission for men in all age categories, except in the old-older group, where the probability of admission was similar to women. Male sex was a minor risk factor for hospitalization (OR 1.37) and mortality (1.14).

ED overcrowding is a problem worldwide. In many countries the number of ED visits is growing faster than the population [31, 32]. In 2012, the ED-HC began implementing a clinical-risk based triage system (Manchester Triage System version II) that assigns categories according to the urgency of the presentation [17]. Cases considered to be non-urgent by the multi-professional team were directed to appropriate alternative primary and secondary care services [33]. The number of cases seen in 2013 decreased significantly in all age-groups except old-older adults. However, the number of admissions, ICU stays and deaths remained stable, as did the number of cases being referred to ED-HC. This indicates that the new triage system was functioning appropriately.

It is important to highlight the growing importance of the oldest patients attending ED. [34–37] In the present study, people aged 80 or over represented 5.1% of ED visits, 9.5% of admissions and 10.1% of ICU admissions. They carried increased ORs for hospital admission (3.49, 95% CI 3.15–3.87), ICU admission (1.27, 1.15–1.39) and in-hospital death (5.93, 5.29–6.66). Between 2009 and 2013, while the three younger age groups selected by the triage system decreased, we observed a stable number of ED visits and increasing ICU admissions by old-older patients. This finding supports the existence of greater risks in this group.

We observed that older patients and those presenting to ED with a referral were more likely to require ICU admission, although the effect size was small in both cases. There were small positive ORs in the two older age-groups (1.19 and 1.27) compared to the reference class of young adults. This finding suggests that differences among age-groups might be mitigated by a judicious selection of patients admitted to ICU. The decision to admit to ICU must weigh up the acuity of illness, existing comorbidities, pre-hospital functional status and the patient’s wishes in relation to resuscitation and ceiling-of-care [38]. In the Netherlands, the number of very elderly patients attending ED has increased, but the number of ICU admissions has remained stable. This is mostly explained by more careful case selection [39]. In Scotland, patients admitted to ICU aged 80–89 had fewer comorbidities than their younger counterparts and underwent a greater proportion of emergency surgeries, but spent less time in ICU than patients under 65 [40]. In the present study, the mean ICU-LOS did not vary significantly between age-groups or sexes, but fell in nearly all the age-groups over the course of the five years studied, suggesting an increased turnover of beds.

This study demonstrates the importance of old-older adults in ED. It highlights the need to identify subgroups that carry greater risk of functional decline and adverse events, such as frail older people, some of whom may be candidates for palliative care. Indeed, subgroups of functional older people at lower risk must also be identified.

## Limitations

We studied a large dataset from a single tertiary Brazilian ED covering a 5-year period. It is important to note that, the care of high-complexity patients is centered in tertiary level hospitals and, when acutely ill, such patients present or are referred to tertiary EDs with profiles similar to ED-HC. To our knowledge, this is the first Latin American comprehensive study to analyze associations between aging and tertiary ED attendance.

Five years is a short period to identify the impact of demographic change in the ED population. Moreover, 7901 ED visits due to 325 individuals were excluded because the cut-off for inclusion was set at 15 ED attendances. The mean age in this group was older (see Table 1). Even with this exclusion, we found an annual increase in the mean and median age, as well as in the proportion of patients aged over 60. We also observed a decrease in the proportion of young adults presenting to the ED.

Changes to the triage system altered the sample composition over the study period. The number of visits fell significantly in 2013, however the number of urgent and complex cases remained stable. Comparing each year to 2009, the effect size was minimal for hospitalization and for ICU admission, and there was no difference in mortality.

The definition of age-groups and classification of reasons for attendance could have introduced misclassification bias. The proportion of younger adults visiting ED-HC decreased annually, whereas those aged over 60 increased. We did not find this pattern when analyzing young-older and old-older groups (see Table 6).

The reasons for attendance were not assigned according to defined criteria and were not recorded by healthcare professionals. However, their inclusion in the study allowed ‘scheduled attendances’ to be identified and excluded. We were also able to differentiate ‘external causes’ (trauma, accidents, poisoning, falls, and so on), ‘general and localized symptoms’, and ‘evaluation requested by another service’ as grouped reported reasons for ED visit.

## Conclusions

Between 2009 and 2013 the proportion of ED visits and admissions by adults aged 60 or over increased in the largest Brazilian tertiary hospital, meanwhile those by young adults fell. Hospitalization, ICU admission and mortality rates increased with older age in both men and women. However, we found similar LOS and ICU-LOS across age-groups, and small effect sizes associated with ICU admission in older patients. Among tertiary ED patients, age is an important risk factor for hospitalization and mortality, but not for ICU admission. Old-older people are at the greatest risk and demand further subgroup stratification.

## Data Availability

The datasets used and/or analysed during the current study are available from the corresponding author on reasonable request.

## Abbreviations

CI: Confidence interval
ED: Emergency Department
GLMM: Generalized Linear Mixed Models
HC: Hospital das Clínicas
ED-HC: Emergency Department associated with the Hospital das Clínicas Central Institute
ICU: Intensive Care Unit
ICU-LOS: length of Intensive Care Unit stay
LAMA: Leave Against Medical Advice
LOS: length of in-hospital stay
LWBS: Leave Without Being Seen
Min-max: minimum-maximum
OR: Odds Ratio
SD: Standard Deviation
SPSS: Statistical Package for Social Sciences
